# Monkey Bites among US Military Members, Afghanistan, 2011

**DOI:** 10.3201/eid1810.120419

**Published:** 2012-10

**Authors:** Luke E. Mease, Katheryn A. Baker

**Affiliations:** US Army Combined Joint Task Force–1, Bagram Air Field, Afghanistan

**Keywords:** animal bite, monkey, Simian, nonhuman primate, rabies, rabies postexposure prophylaxis, herpes B virus, Macacine herpesvirus 1, tetanus, military, Afghanistan, B-virus, viruses, zoonoses

## Abstract

Bites from *Macaca* *mulatta* monkeys, native to Afghanistan, can cause serious infections. To determine risk for US military members in Afghanistan, we reviewed records for September–December 2011. Among 126 animal bites and exposures, 10 were monkey bites. Command emphasis is vital for preventing monkey bites; provider training and bite reporting promote postexposure treatment.

## Abstract 2

If you were to list all the dangers faced by US military personnel serving in Afghanistan, your list would be long, but would it include monkey bites? It should. The US Army recently examined this risk and found that in just 4 months, 10 service members were bitten by monkeys. And there may have been more, unreported, bites. Most monkeys were pets owned by Afghan National Security Forces and Afghan civilians, so the risk of being bitten could increase as US forces work more closely with these Afghan people. Monkey bites can spread rabies, tetanus, or other bacterial infections, or B-virus infection to humans. Bites can be minimized by enforcing military policies that prohibit pet adoption and animal contact, and secondary infections can be reduced by providing better training to military health care providers on how to treat animal bites.

Military members deployed to Afghanistan face many risks; among these are bites from *Macaca* *mulatta* monkeys and possible subsequent infections. In August 2011, a 24-year-old US Army soldier died of a rabies infection contracted while in eastern Afghanistan. This tragedy highlights the threat that animal bites pose to deployed military members.

During 2001–2010, a total of 643 animal bites among deployed US military members were reported (*1*). Dogs were implicated in 50% of these bites, but several other animals pose risk as well. Prominent among these is the nonhuman primate *M. mulatta* (rhesus macaque), native to and commonly kept as a pet in Afghanistan (*2*) (Figure). Risks from *M.* *mulatta* monkey bites include physical trauma and/or infection with B-virus (Macacine herpesvirus 1), oral bacteria (including *Clostridium tetani*), and rabies virus. Although not well characterized in Afghanistan, the risk for exposure to *M.* *mulatta* monkeys has been described (*3*) for researchers (*4*), tourism workers (*5*), and US pet owners (*6*). We examined this risk for US military members deployed to eastern Afghanistan. The work presented herein was reviewed and deemed exempt from internal review board oversight by the Joint Combat Casualty Research Team, the human subjects review board responsible for oversight of human subjects research affecting US military members in Afghanistan.

**Figure:**
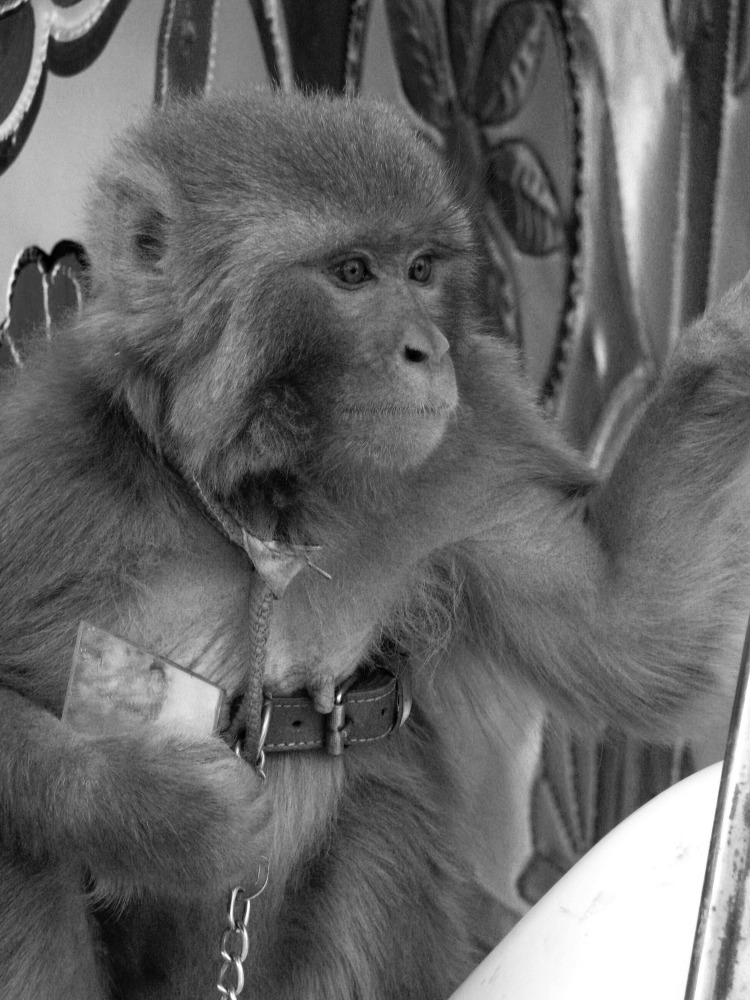
Pet monkey (*Macaca mulatta*), Afghanistan, 2011. Photograph courtesy of Ronald Havard.

## The Study

Information about all reported animal bites and exposures affecting US military and coalition personnel is collected by preventive medicine officers assigned to Combined Joint Task Force–1 in eastern Afghanistan. We evaluated these records to identify and describe monkey bites and high-risk exposures among US military members serving in eastern Afghanistan during September–December 2011. For this study, eastern Afghanistan refers to North Atlantic Treaty Organization Regional Command East, which covers ≈43,000 square miles (110,000 km^2^). The US military population in eastern Afghanistan during the study period was ≈23,500 persons. Case information obtained included patient age, sex, rank, branch of military service, animal exposures, and treatment details.

We evaluated the cases for the 5 parameters that comprise appropriate initial treatment according to the literature. The parameters are wound care (appropriate cleansing of the wound) (*7*), antiviral medications for B-virus (valacyclovir) (*8*), antimicrobial drugs for oral bacteria (amoxicillin/clavulanic acid or clindamycin plus sulfamethoxazole/trimethoprim) (*3*), verification of up-to-date tetanus vaccination status or vaccine administration in accordance with Advisory Committee on Immunization Practices guidelines (*9*), and rabies postexposure prophylaxis (PEP). US military policy advised that rabies PEP should adhere to World Health Organization guidelines (*10*), which recommend giving human rabies immunoglobulin plus 5 doses of rabies vaccine. In accordance with the same policy, adherence to Advisory Committee on Immunization Practices guidelines for rabies PEP with human rabies immunoglobulin plus 4 doses of rabies vaccine was also acceptable (*11*).

When appropriate initial treatment was not administered, subsequent follow-up was conducted to ensure that patients received required treatment. Appropriate treatment was accomplished by contacting and coordinating with the responsible provider, the patients, and their commanders.

During the study period, we identified 126 cases of animal bites or serious exposures (involving animal neural tissue or saliva affecting the mucosal surfaces or open wounds of the patient). Among these cases, 10 were cases of monkey bites.

Among the 10 military members who had been bitten by monkeys, age range was 22–44 years (Table); most (7) were <30 years of age, and 8 were male. All were junior enlisted or noncommissioned officers; 8 were members of the Army, and 2 were members of the Air Force (Table).

**Table:** Characteristics of US military members bitten by monkeys, eastern Afghanistan, September–December, 2011*

Patient no.	Age, y/sex	Military branch	Treatment received					Monkey ownership
			Wound care	Valacyclovir	Antimicrobial drug	Tetanus vaccine	Rabies vaccine, HRIG	
1	39/M	Army	–	+	+	+	+	ANSF
2	27/M	Army	+	+	+	+	+	CIV†
3	22/M	Army	–	+	+	–	+	CIV
4	44/F	Army	+	–	+	–	–	CIV
5	31/M	Army	+	–	+	+	+	ANSF
6	26/M	Air Force	+	–	–	–	–	US military
7	26/M	Army	–	+	–	–	+	ANSF
8	27/M	Army	+	–	+	+	+	ANSF
9	22/M	Army	–	–	+	–	+	Unknown
10	25/F	Air Force	+	+	+	–	+	Unknown

*HRIG, human rabies immunoglobulin; –, not administered; +, administered; ANSF, Afghan National Security Forces; CIV, Afghan civilian. †Monkey euthanized. Brain, tested at US Army Veterinary Laboratory Europe, was negative for rabies and B-virus.

In terms of treatment, 6 received appropriate wound care and washing, 5 received appropriate B-virus prophylaxis, and 8 received appropriate antimicrobial drugs (Table). In terms of prophylaxis, only 4 were evaluated for tetanus status, and 8 received appropriate rabies PEP. Beyond the initial trauma and follow-up visits for rabies PEP, no visits for any illness possibly associated with the bite or exposure were recorded.

All cases involved different monkeys, 8 of which were kept as pets. Of these 8 pet monkeys, 4 belonged to Afghan National Security Forces (ANSF), 3 belonged to Afghan civilians, and 1 belonged to US military members. For the other 2, no ownership data were available; they could have been wild or pets. One monkey was euthanized and sent to US Army Veterinary Laboratory Europe for testing; brain samples were negative for rabies and B-virus.

## Conclusions

Our identification of 126 reported bites or exposures over just 4 months suggests that the 643 animal bites reported for all deployed US military members for the past decade greatly underestimate the true number of animal bites in this population. The number of bites and exposures identified in this study might represent more accurate reporting because of increased attention to animal bites after the US soldier died in August 2011. It is possible that before that time, only more severe bites and exposures were reported but that after that time, more lower-risk exposures might have been reported.

The risk for monkey bites in other populations has been described. The 10 monkey bites reported in this study demonstrate that US and coalition military members in Afghanistan are also at risk for the trauma and the B-virus, bacterial, tetanus, and rabies infections that can result from monkey bites and exposures. The demographics of the population bitten (Army, age <30 years, and male) is representative of the underlying population at risk.

Most monkey-bite patients received appropriate care. This care is laudable, considering the recognized difficulties in treating monkey bites (*12*). Some patients, however, did not receive appropriate medical treatment initially. Because treatment of monkey bites is not a standard part of US medical education, inadequate treatment could reflect insufficient training and lack of familiarity among US-trained health care providers. It is imperative that before providers are deployed to Afghanistan, they receive proper instruction on the care of animal bites and exposures. Appropriate reporting of any animal bite to military preventive medicine personnel is crucial because it permits oversight of care and timely correction of deficiencies.

Most (7/10) monkeys involved were pets owned by ANSF or Afghan civilians. As the mission in Afghanistan shifts from combat to ANSF mentoring and reconstruction, US and coalition troops will come into increasingly close contact with ANSF and Afghan civilians. Accordingly, the likelihood of deployed US military members being exposed to monkeys in Afghanistan will probably increase. However, although risk for contact with monkeys might increase, an increase in bites is not inevitable. Explicit orders prohibit deployed US military members from adopting local mascots and from interacting with animals or pets owned by ANSF or Afghan civilians. To mitigate the risk for animal bites, it is crucial that commanders enforce these regulations (*13*).

The risk of being bitten by a monkey could increase as US forces work more closely with ANSF and Afghan civilians. Bites could be prevented by appropriate emphasis from command and enforcement of existing policies prohibiting pet adoption and animal contact. Treatment of patients who are bitten could be improved by further training of military health care providers on appropriate treatment for animal bites, including monkey bites.
